# A study on collaborative governance of excessive medical care based on three-way evolutionary game and simulation

**DOI:** 10.3389/fpubh.2025.1593398

**Published:** 2025-07-17

**Authors:** Hanxiang Gong, Tao Zhang, Xi Wang, Baoling Wu, Shufang Zhao

**Affiliations:** ^1^Faculty of Humanities and Social Sciences, Macau Polytechnic University, Macao, Macao SAR, China; ^2^The Second Affiliated Hospital, Guangzhou Medical University, Guangzhou City, Guangdong Province, China

**Keywords:** excessive medical care, collaborative governance, evolutionary game, simulation analysis, healthcare regulation

## Abstract

**Introduction:**

Although China has made some progress in regulating and governing overtreatment behaviors in healthcare institutions, excessive medical care remains a persistent challenge in the Chinese healthcare sector.

**Methods:**

This study adopts a perspective of bounded rationality and employs evolutionary game theory to construct a collaborative governance model involving government regulatory departments, healthcare institutions, and patients. The model analyzes the strategic stability of each participant and examines the impact of various factors, such as fiscal subsidies, government fines, rectification costs, regulatory costs, reasonable treatment income, and overtreatment income, on the strategic choices of the game participants. Parameter sensitivity within the three-party gaming system is also investigated through simulation analysis.

**Results:**

The findings indicate that when patients trust treatment outcomes and healthcare institutions are more inclined to provide appropriate care, government regulatory departments tend to adopt a more relaxed regulatory strategy. Simulation results show that increasing government fiscal subsidies, raising reasonable treatment income, and strengthening supervision and rectification efforts are effective in reducing overtreatment behaviors.

**Discussion:**

The decision-making of government regulatory departments is influenced by the degree of patient trust. Improving collaborative governance for overtreatment requires establishing comprehensive laws and regulations, leveraging government regulatory functions, strengthening internal constraint mechanisms in healthcare institutions, and raising patients' awareness of their rights and supervisory responsibilities.

## 1 Introduction

Despite these insights, there are notable research gaps. First, most studies focus on the governance of overtreatment behaviors by either government regulatory departments or individual medical institutions, without considering the interactive dynamics among these stakeholders. Second, the current research methods primarily utilize static case analyses, neglecting the dynamic evolution of repeated interactions among multiple agents. This limitation hinders the development of comprehensive and sustainable governance strategies. Addressing these gaps, our study employs evolutionary game theory to explore the collaborative governance of overtreatment, considering the dynamic interplay among government regulatory departments, medical institutions, and patients. This approach provides a novel perspective and practical guidance for formulating long-term solutions to overtreatment issues in the healthcare sector.

This article adopts an evolutionary game theory perspective to construct a tripartite evolutionary game model involving government regulatory agencies, medical institutions, and patients. The model analyzes the strategic stability of each game participant and explores the impact of various factors, such as fiscal subsidies, government fines, and medical institution income, on their strategic choices. We also use simulation analysis to test the model under different initial conditions, providing valuable insights for policy formulation to reduce overtreatment.

Internationally, the issue of overtreatment has been widely studied across various healthcare systems. For example, in the United States, research has highlighted the financial incentives driving unnecessary medical services, leading to increased healthcare costs and patient harm. Studies in Europe have focused on the role of defensive medicine, where doctors order excessive tests and procedures to avoid litigation. In Australia, overdiagnosis in cancer screenings has been identified as a significant problem, leading to unnecessary treatments and patient anxiety. Furthermore, global initiatives such as the *Choosing Wisely* campaign, launched by the American Board of Internal Medicine Foundation in 2012, have played a significant role in shaping global discourse on unnecessary medical interventions. This movement encourages physicians and patients to engage in shared decision-making and avoid tests and procedures that are unlikely to benefit the patient. As of 2024, the campaign has been adopted by over 25 countries, influencing national guidelines and promoting value-based care. Its widespread adoption highlights a global consensus on the urgency of reducing overtreatment, and serves as a conceptual foundation for collaborative governance models like the one proposed in this study. Including such perspectives helps bridge international best practices with localized healthcare governance challenges in China (1, 2).

China's healthcare system has transitioned from a planned economy to a market economy since 1985. Public hospitals, which accounted for 84.0% of outpatient visits and 80.8% of hospital admissions in 2020, face financial challenges due to limited government subsidies. For example, in 2019, fiscal donations accounted for only 11.1% of the total income of public hospitals nationwide, while personnel expenses accounted for as much as 36% of total public hospital expenditures. To maintain operations, some hospitals rely on revenue from drugs, consumables, and examinations, often linking revenue performance to doctors' salaries, leading to a situation where it is difficult to fundamentally change the practice of “sustaining healthcare with drugs.” At the same time, in an increasingly competitive healthcare service market, hospitals adopt measures such as expanding scale, updating equipment, and introducing new diagnostic and treatment technologies to increase revenue. These substantial medical investments inevitably need to be realized by collecting patient fees for medical services. Overtreatment issues in the Chinese medical market have become increasingly prominent (3), with various manifestations, including excessive examinations (4, 5), overmedication (6, 7), overtreatment (8, 9), and induced surgeries (10, 11).

Numerous Chinese scholars have extensively explored the factors contributing to overtreatment behaviors and proposed solutions. For instance, some recent empirical studies have used micro-level data to examine overtreatment behaviors in Chinese hospitals. These studies have investigated the impact of compensation mechanism reforms, such as canceling drug markups or controlling the proportion of drug costs, on medical expenses (12–14). The literature indicates that these policies have significantly reduced the proportion of drug costs. However, the proportion of medical service costs has continued to rise, and patients' total medical expenditures have not decreased (15, 16). This indirectly reflects that hospitals and doctors have significant information advantages in medical consumption and use this advantage for overtreatment.

China's healthcare reforms, aimed at supervising and rectifying overtreatment, have yielded measurable progress with the curtailment of the rapid growth of medical expenses and the restructuring of hospital revenue sources, as public hospital revenue growth rates fell from 20% to 12.6% from 2011 to 2019, drug revenue proportions decreased from 44.8% to 31.0% between 2012 and 2020, and the ratio of personnel expenditures rose from 24% to 36%, alongside a reduction in the personal health expenditure share from 35.3% to 27.7% from 2010 to 2021 (17). However, despite these advancements, overtreatment persists in critical areas such as cancer treatment. For example, according to the National Health Commission, cancer treatment costs have increased by 15% annually from 2015 to 2020, yet the mortality rate for major cancers has not shown a commensurate decrease, remaining relatively stable around 15% over the same period. This discrepancy underscores the systemic imperfections and highlights the urgent need for further reforms. These challenges necessitate a deeper engagement with multi-entity collaborative governance and significant innovation in medical supervision to propel public hospital reforms, enhance medical professionalism, and intensify public health education (18–20). Our study, informed by the concept of eco-generativity and the collaborative ethos it espouses (21–23), contributes to this complex governance landscape by applying an evolutionary game theory approach to examine the interplay between government, healthcare institutions, and patients, enriching the discourse on effective governance strategies in healthcare and beyond.

This article will adopt an evolutionary game theory perspective to construct a tripartite evolutionary game model involving government regulatory agencies, medical institutions, and patients. We will analyze the strategic stability of each game player and the impact of various factors on their strategic choices. Furthermore, we will use Matlab 2020b for simulation analysis to verify the effectiveness of the model analysis under different initial conditions. The motivation behind employing the evolutionary game approach in this study lies in its ability to effectively model the complex interactions among government regulatory departments, healthcare institutions, and patients in the context of addressing overtreatment. With overtreatment being a persistent and multifaceted issue, the evolutionary game theory allows us to explore long-term behavior, strategic stability, and decision-making processes influenced by bounded rationality. This approach enables us to simulate various scenarios and assess the effectiveness of collaborative governance strategies, providing evidence-based policy recommendations for reducing overtreatment in healthcare institutions.

In addressing the persistent issue of excessive medical care, existing literature predominantly focuses on singular factors, such as policy impacts, patient behaviors, or hospital management strategies, often overlooking the complex interplay among government, healthcare institutions, and patients. This study introduces an evolutionary game model encompassing these three stakeholders, thereby bridging a significant gap in the literature. Our approach not only analyzes the stability of strategies among these parties but also simulates the impact of various factors on strategic choices, offering novel insights and practical guidance for the collaborative governance of excessive medical care. Moreover, the sensitivity analysis of parameters conducted herein furnishes a scientific basis for policymaking. Evolutionary game theory is particularly pertinent for this analysis due to its capacity to simulate and predict the behaviors of different stakeholders under various policy and economic scenarios. This allows us to explore how factors like subsidies, fines, and healthcare costs influence decision-making processes in medical care, thereby enriching the theoretical foundation and suggesting effective mechanisms for the rational utilization of medical resources and enhancement of service quality. Therefore, this research fills a critical void in the literature by offering a unique and comprehensive model to understand and address the multifaceted issue of overtreatment in healthcare.

## 2 Literature review

### 2.1 Reasons for the formation of overmedication

The formation of medical overuse can be attributed to several dimensions, among which the medical system and economic incentives are key factors. In some countries, doctors and hospitals increase their income by providing more medical services, a payment model that easily leads to medical overuse. Strockbine et al. (24) found that continuity of medical services is significantly associated with a reduction in the potential overuse of procedures, indicating that high-continuity care helps reduce medical overuse. Additionally, Zhou et al. (25) pointed out that the supply of regional medical resources is significantly associated with systemic overuse, especially in areas with lower densities of primary care physicians. On the other hand, the doctor-patient relationship and patient expectations are also important factors influencing medical overuse. Jankauskaite et al. (26) surveyed and found that 83% of responding doctors believed that patient and family expectations are major drivers of overtreatment. Patients often expect more tests and treatments for psychological reassurance, which can prompt doctors to perform unnecessary medical procedures.

Strict enforcement of clinical guidelines and defensive medicine are also contributing factors to medical overuse. Scott (27) reported that clinical audits in Australia showed overuse rates above 30% for coagulation tests, blood cultures, and troponin assays. To avoid potential legal actions, doctors often take defensive medical measures, conducting unnecessary tests and treatments, thus leading to medical overuse. Furthermore, the unequal distribution of medical resources is a significant cause of medical overuse. Ahn et al. (28) found that older individuals, the unemployed, and those with higher education levels among medical aid beneficiaries were more likely to belong to the overuse group. Medical institutions in resource-rich areas are more prone to medical overuse, whereas those in resource-poor areas may face issues of insufficient medical services.

Overmedicalization is a significant issue in China. Patients' lack of medical knowledge and pursuit of expensive imported drugs lead to inappropriate treatments. Government financial support for public hospitals has decreased, and the 2017 healthcare reform's zero-markup policy on drugs has reduced hospital revenue without effective compensation mechanisms. Hospitals increase revenue through excessive testing and repeated treatments. To avoid medical disputes, doctors often require comprehensive examinations, further exacerbating overmedicalization.

### 2.2 Negative consequences caused by excessive medical treatment

Medical overuse leads to resource waste, increased economic burden, heightened health risks for patients, reduced treatment effectiveness, and negative environmental impacts. Scott (27) found that over 30% of medical procedures in Australian hospitals are excessive, wasting valuable medical resources. Zhou et al. (25) highlighted that regional overuse of medical resources in the United States leads to significant economic costs. Thiel and Richie (29) emphasized that medical overuse in the U.S. generates approximately 479 million metric tons of carbon dioxide emissions annually, increasing health risks and environmental burdens. Weaver et al. (30) indicated that overuse of proton pump inhibitors in intensive care units can lead to unnecessary side effects and health risks. Jankauskaite et al. (26) found that pediatricians in several European countries identified high expectations from parents and patients as a primary driver of medical overuse, leading to unnecessary diagnostics and treatments. Strockbine et al. (24) demonstrated that high continuity of care can reduce medical overuse, improving patient satisfaction and treatment outcomes.

### 2.3 Progress of research on overmedication

Significant progress has been made in international research on irrational diagnosis and treatment or overdiagnosis, involving areas such as the overuse of medical services, low-value services, drug management, shared decision-making, clinical decision support systems, and the application of evidence-based medicine. Researchers have proposed various methods and strategies to reduce the incidence of irrational diagnosis, treatment, and overdiagnosis to improve the efficiency and quality of medical services. These studies provide valuable references for policymakers and clinicians alike (24, 31–33).

Berwick (34) proposed the “Third Era” to advocate for a change in healthcare service models to improve efficiency and quality. Colla et al. (35) conducted a systematic review of interventions to reduce the use of low-value healthcare services. Doust et al. (36) proposed a checklist for modifying disease definitions. Durand et al.'s (37) systematic review and meta-analysis assessed the impact of interventions supporting shared decision-making in reducing health inequalities. Kelly et al. (38) reviewed the experience of the UK's National Institute for Health and Clinical Excellence (NICE) in developing public health guidelines. Califf et al. (39) emphasized the utilization of big data, digital medical records, and better collaboration to improve the quality and efficiency of evidence generation, in order to better support healthcare policy and practice and thus reduce irrational medical issues. Wimpenny and Kirkpatrick (40) suggested adopting technology-supported drug management systems and strengthening the training of medical professionals to improve the efficiency and safety of drug management. These articles all address issues of irrational diagnosis and treatment or overdiagnosis. Morgan et al. (41, 42) reviewed the latest developments in overdiagnosis and proposed a practical framework for understanding and reducing medical overuse. Brownlee et al. (43) investigated the overuse of medical services on a global scale. Schwartz et al. (44) studied changes in low-value services in the US Medicare program. Raja et al. (45) conducted a randomized controlled trial examining the impact of computerized clinical decision support systems on emergency department abdominal pain diagnostic imaging to reduce misdiagnosis or irrational diagnosis and treatment occurrences.

Despite this progress, a critical research gap remains concerning collaborative governance to address excessive medical care comprehensively. Previous research has primarily focused on isolated interventions and static case analyses, overlooking the complexity of the multi-stakeholder environment involved in addressing overtreatment. An in-depth exploration of multi-entity collaborative governance is necessary to develop a holistic solution to tackle overtreatment behaviors effectively.

### 2.4 Stakeholders' game in overmedication

The essence of game theory is the mutual competition and cooperation among participants in aspects such as interest distribution, risk-taking, and responsibility-sharing. In the issue of overdiagnosis and overtreatment, a game relationship exists among government regulatory departments, medical institutions, and patients (46–48). Firstly, government regulatory agencies face a dilemma between controlling medical costs and ensuring the quality of medical care. Zhou et al. (25) indicated that regions with a higher density of primary care doctors exhibit less systemic medical overuse, suggesting that the rational allocation of medical resources can reduce overuse, which requires a balance of policy support and regulatory efforts. Secondly, medical institutions face a conflict between pursuing economic benefits and providing high-quality medical services. The fee-for-service model incentivizes doctors to increase the number of treatments to raise income, leading to medical overuse. Scott (27) found that more than 30% of medical procedures in Australian hospitals are excessive, reflecting the behavior of medical institutions under economic pressure. Lastly, high patient expectations and demands are also significant factors driving medical overuse. Jankauskaite et al. (26) reported that pediatricians in several European countries believe that high expectations from parents and patients are the main drivers of unnecessary diagnoses and treatments. The game between patient expectations and doctors' professional judgment exacerbates the phenomenon of medical overuse.

### 2.5 Evolutionary game theory in healthcare governance: recent applications

In recent years, evolutionary game theory has increasingly been applied to analyze complex, dynamic decision-making processes in healthcare governance, particularly in contexts characterized by bounded rationality, multi-stakeholder interactions, and information asymmetry. Unlike static optimization models, evolutionary game theory captures the learning behaviors and adaptive strategies of various actors, including governments, medical institutions, and patients, as they respond to policy interventions and incentives over time.

Tong et al. (49) utilized an evolutionary game framework to explore regulatory strategies for medical devices within healthcare delivery systems, highlighting the role of government subsidies and institutional compliance in optimizing resource allocation. Similarly, Yue et al. (50) developed a tripartite game model involving government, private institutions, and patients to study public-private partnerships (PPPs) in elderly care, demonstrating how credibility and penalty mechanisms influence long-term stakeholder behavior. In the context of technological healthcare transformation, Yang and Wang (51)investigated AI-driven elderly care adoption, showing that bounded rationality and trust play crucial roles in stakeholder evolution, while Bai et al. (52) applied evolutionary games to assess the risks and benefits of health data sharing, illustrating how cybersecurity breaches and patient trust dynamically impact data policies. Several China-based empirical studies have also contributed to this field. Zhao et al. (67) constructed an evolutionary game model on cross-regional cancer treatment, integrating patient mobility and treatment quality. Du et al. (53) analyzed stakeholder interactions in remote diagnosis systems using a replicated dynamics approach, revealing how local government subsidies can shift strategies from cost-saving to service-enhancing. Overall, recent works converge on a shared insight: effective healthcare governance must be adaptive and co-evolutionary, responding dynamically to shifting incentives, risk perceptions, and institutional trust (54). These findings strongly support the use of evolutionary game theory as both an analytical lens and a simulation tool to test policy interventions in real-world healthcare systems.

## 3 Evolutionary model assumptions and model description

### 3.1 Model description

In the collaborative governance of medical overuse, the government regulatory authorities can adopt two distinct strategies: strict regulation and loose regulation. Strict regulation involves imposing fines or other sanctions on institutions that engage in excessive treatment, providing a strong deterrent against such behavior. Furthermore, it involves imposing severe penalties for irrational medical practices. This regulatory approach aims to safeguard the public interest, enhance healthcare quality, and prevent the occurrence of medical overuse.

In contrast, loose regulation adopts a more flexible and lenient management approach, allowing medical institutions greater autonomy and decision-making space. However, this approach may lead to a need for standardized practices and constraints in the behavior of medical institutions, thereby increasing the risk of patients receiving unnecessary medical services. Some medical institutions, driven by profit motives, may engage in excessive medical treatments, imposing undue medical burdens and risks on patients. As a result, when choosing a regulatory strategy, government regulatory authorities need to carefully weigh and balance the autonomy of medical institutions with the interests of patients to ensure the realization of healthcare quality and public welfare. Based on this, the paper establishes a tripartite game model for governing medical overuse and its variables, as shown in Table 1.

**Table 1:** Variables and variable meanings.

**Gaming subjects**	**Variables**	**Definitions of variables**	**Remarks**
Patients	*C*_*m*_	Cost of overmedicalization	Costs incurred from diagnosis and treatment exceeding the actual needs of the disease
	*C*_*t*_	Benefits of appropriate diagnosis and treatment	Benefits of patients receiving appropriate diagnosis and treatment
	*C*_*P*_	Probability of patient complaints	Probability of patient questioning and lodging complaints against healthcare institutions
	*C*_*i*_	Compensation income	Compensation income for patients affected by excessive medical treatment
Medical institutions	*W*_*m*_	Income from overmedicalization	Income obtained by hospitals from excessive medical treatment of patients
	*W*_*t*_	Income from appropriate diagnosis and treatment	Income generated by healthcare institutions from providing appropriate diagnosis and treatment
	*G*_*f*_	Government fines	Government fines imposed on healthcare institutions
	*G*_*s*_	Government financial subsidies	Government financial subsidies to healthcare institutions
	*E*_*r*_	Benefits from good reputation	Positive reputation benefits resulting from the provision of appropriate diagnosis and treatment
	*O*_*c*_	Operational costs of hospital medical services	Costs associated with daily medical practices of healthcare institutions
Government regulators	*E*_*g*_	Social benefits	Social benefits derived from healthcare institutions adhering to rules, accepting supervision by the government
	*C*_*r*_	Regulatory costs	Costs of government oversight and supervision
	*G*_*g*_	Remediation costs	Costs incurred by the government in addressing the medical practices of healthcare institutions
	*P*_*g*_	Government credibility	Reputation damage resulting from inadequate government regulation

While the primary focus of our simulation model is on the issue of overtreatment in healthcare, its design incorporates fundamental principles of strategic interaction and decision-making that are applicable to a wider range of medical overuse issues. This adaptability is rooted in the model's ability to simulate varying scenarios and stakeholder behaviors, making it a valuable tool for exploring different types of medical overuse beyond the specific context of this study. By adjusting the parameters and variables, researchers and policymakers can use this model to analyze other medical overuse scenarios, providing a versatile framework for understanding and addressing these challenges.

In constructing the three-party evolutionary game model and conducting simulation analysis, all key variables listed in Table 1 were quantified based on national statistics, policy documents, industry reports, and relevant literature. This approach ensures both empirical relevance and computational feasibility. For monetary variables—such as government subsidies, fines, operating costs, reasonable treatment income, and overtreatment income—we referred to sources including the *China Health and Wellness Statistical Yearbook 2022* and bulletins from the *National Healthcare Security Administration*. These values were scaled proportionally within the simulation. For non-monetary variables—such as Social Benefits (*E*_*g*_), Government Credibility (*P*_*g*_), Patient Complaint Probability, and Reputation Gains—direct empirical values are not available. Therefore, they were normalized on a [1–10] scale, based on expert judgment, literature references, and policy interpretations. Sensitivity analyses (Section 5.2) were conducted to ensure model robustness across variable ranges. All parameter values and initial strategy settings (x = 0.5, y = 0.5, z = 0.5) are detailed in Section 5.1 to support reproducibility and model transparency.

It should be noted that, due to data access constraints, some variables in this study—particularly those related to stakeholder behavioral probabilities or non-monetary payoffs—are difficult to obtain directly from public databases or real-world hospital surveys. Consequently, our simulation parameter settings are based on the best available official statistics, policy documents, and expert opinions. Although this inevitably introduces some limitations, it is in line with the prevailing practice in the field for theoretical modeling studies. The combination of transparent parameter documentation and extensive sensitivity analysis aims to maximize the empirical relevance and robustness of our findings in the absence of complete real-world datasets.

### 3.2 Evolutionary model assumptions

In this study, the three major game players, government regulatory agencies, medical institutions, and patients, pursue their interests to the greatest extent possible and need to decide their strategies based on the choices of the other two parties. This game is not a traditional static one-time game but rather a process in which each boundedly rational individual within the three major game groups adjusts their strategy based on their own experience, ultimately leading to stability. In constructing the game model and analyzing the stability of strategies, equilibrium points, and the interplay of various factors among the participants, this study posits the following seven assumptions, grounded in the empirical evidence and theoretical constructs from evolutionary game theory literature within healthcare and broader contexts. The assumptions draw on the dynamics of administrative and medical institutions as highlighted by Xu et al. (55), the collaboration among healthcare and social care outlined by Sun et al. (56), and the sustainable innovation strategies among stakeholders discussed by Lu et al. (57). Additionally, they are informed by the work on differential game models and coordination within green supply chains by Mohsin et al. (58), which offer parallels to the administrative dynamics within healthcare systems. The behavioral strategies of manufacturing firms in response to government participation, explored by Shi and Su (59), serve as analogies for interactions within healthcare organizations. The approach of Chu et al. (60) in utilizing policy simulation to engage multiple players in evolutionary games informs our understanding of public participation in healthcare governance. The multi-stakeholder perspective provided by Hati et al. (61) from the sharing economy literature, along with the community proactive health management model by He and Wang (62), are integrated to reflect the complexity and diversity of participant dynamics specific to the context of overtreatment in the Chinese healthcare system. These assumptions encapsulate the essence and findings from these seminal works.

In constructing the tripartite game model, this study adopts evolutionary game theory (EGT) rather than alternative modeling frameworks such as system dynamics, complex networks, or principal-agent models, due to the following reasons:

First, EGT assumes bounded rationality and emphasizes how agents adjust their strategies over time through imitation and adaptation in a context of incomplete information. This closely aligns with the behavioral characteristics observed in the interactions among government regulators, profit-driven healthcare institutions, and patients with limited oversight capabilities. In contrast, classical static game models may fail to capture the evolving nature of stakeholder strategies in repeated interactions, while system dynamics focuses more on variable feedback loops than on strategic behavior among heterogeneous agents. Second, EGT has been successfully applied in various public policy and healthcare contexts. For instance, Xu et al. (55) and Sun et al. (56) utilized EGT to analyze collaborative mechanisms in healthcare regulation and social care integration, highlighting the effectiveness of adaptive strategies. Lu et al. (57) extended this approach to sustainable supply chain governance, demonstrating its generalizability and robustness in multi-agent coordination problems. In comparison, principal-agent models focus on incentive alignment under asymmetric information but are typically limited to dyadic interactions (e.g., government–hospital or hospital–patient), lacking the structural flexibility and evolutionary dynamics required to model three-party interactions in complex healthcare ecosystems. Therefore, EGT offers a more suitable and dynamic framework for simulating the evolving strategies of all three stakeholders and deriving actionable policy insights from their interactions.

#### 3.2.1 Assumption 1

Participating entities. The regulation and rectification of overtreatment require the joint participation of government health regulatory agencies, medical institutions, and patients. This study constructs a tripartite evolutionary game model consisting of government regulatory agencies, medical institutions, and patients, with all three parties making strategic choices under bounded rationality. However, due to information asymmetry, patients are at a disadvantage and have limited ability to influence the decisions of hospitals and the government. Patients often rely on trust in healthcare providers and may find it difficult to discern over-examination or overtreatment. Despite this, patients remain a critical part of the game as their trust and choices contribute to the overall dynamics, even if their strategic influence is more passive compared to hospitals and government agencies.

#### 3.2.2 Assumption 2

Decision-making of each stakeholder. The three main entities, government regulatory agencies, medical institutions, and patients, each have two strategic choices: patients can choose to recognize the results of treatment /not recognize the results of the treatment, with probabilities of *x* and 1−*x*, respectively; medical institutions can choose overtreatment/reasonable treatment, with probabilities of *y* and 1−*y*, respectively; government regulatory agencies can choose strict supervision/relaxed supervision for unreasonable treatment behavior, with probabilities of *z* and 1−*z*, respectively. Individuals in the three entities possess bounded rationality and will adopt imitative strategies to choose their own strategies based on the choices of other individuals within their group.

#### 3.2.3 Assumption 3

In this study, we assume that the relationship between doctors and medical institutions is a traditional principal-agent relationship, meaning the behavior of doctors is consistent with that of medical institutions. The revenue from overtreatment (*W*_*m*_) is greater than the revenue from reasonable treatment (*W*_*t*_).

#### 3.2.4 Assumption 4

When medical institutions implement reasonable treatment, their reasonable treatment revenue is *W*_*t*_, and the government will provide financial subsidies *G*_*s*_. At the same time, they will generate a good reputation benefit *E*_*r*_. When medical institutions carry out overtreatment, their overtreatment revenue is *W*_*m*_, and the government regulatory department will impose fines, thereby reducing the overall revenue of these institutions *G*_*f*_. This penalty serves as a direct deterrent to excessive medical practices. Additionally, the government may reduce or eliminate financial subsidies, creating a dual mechanism of punishment and incentive to promote compliance with reasonable treatment practices. Regardless of whether medical institutions choose reasonable treatment or overtreatment, they will incur medical operation costs.

#### 3.2.5 Assumption 5

When medical institutions implement overtreatment and face patient complaints, they will generate regulatory costs *C*_*r*_ and rectification costs *G*_*g*_, and reduce government credibility *P*_*g*_.

#### 3.2.6 Assumption 6

Each stakeholder is a participant with bounded rationality. Since different game participants hope to maximize their expected benefits under the premise of information asymmetry, their strategic choices gradually evolve over time and stabilize at the optimal strategy, achieving collaborative governance. The stable strategy is not achieved overnight. However, due to the information asymmetry between hospitals and government regulatory agencies, it is often difficult for the government to detect excessive treatment behaviors directly. To address this, the government employs several mechanisms such as healthcare insurance audits, random inspections, and patient complaints to indirectly monitor and detect these behaviors. Furthermore, emerging technologies like big data and AI are increasingly being used to enhance the detection process.

Parameters are set according to the model assumptions, as shown in Table 1.

## 4 Evolutionary model construction and analysis

### 4.1 Model construction

Based on the aforementioned assumptions and variable definitions, a mixed-strategy payoff matrix is established for the government regulatory department, medical institutions, and patients, as shown in Table 2.

**Table 2:** Matrix of mixed strategy game among government regulators, medical institutions and patients.

**Stakeholders / Actions**	**Healthcare Institutions**	**Government regulators**	
		**Strict regulationz**	**Loose regulation** **1−z**
Patients recognize the results of treatment *x*	Medical institutions reasonable treatment 1−*y*	*C*_*p*_+*C*_*i*_−*C*_*m*_	*C*_*m*_
		*W*_*m*_−*G*_*f*_−*O*_*c*_	*W*_*m*_+*G*_*s*_−*O*_*c*_
		−*C*_*r*_−*G*_*g*_−*P*_*g*_	*P*_*g*_
	Excessive medical treatment in medical institutions *y*	*C*_*t*_	*C*_*t*_−*C*_*p*_−*C*_*i*_
		*W*_*t*_+*G*_*s*_+*E*_*r*_−*O*_*c*_	*W*_*t*_+*G*_*s*_+*E*_*r*_
		*E*_*r*_−*C*_*r*_−*G*_*g*_	*E*_*g*_
Patients do not recognize the results of the treatment 1−*x*	Medical institutions reasonable treatment 1−*y*	*C*_*p*_+*C*_*i*_−*C*_*m*_	−*C*_*m*_−*C*_*p*_−*C*_*i*_
		*W*_*m*_−*G*_*f*_−*O*_*c*_	*W*_*m*_−*O*_*c*_
		*P*_*g*_−*C*_*r*_−*G*_*g*_	−*E*_*g*_−*P*_*g*_
	Excessive medical treatment in medical institutions *y*	*C*_*t*_+*C*_*p*_+*C*_*i*_	*C*_*t*_−*C*_*p*_
		*W*_*t*_+*G*_*s*_+*E*_*r*_−*O*_*c*_	*W*_*t*_+*G*_*s*_+*E*_*r*_−*O*_*c*_
		*E*_*g*_−*C*_*r*_−*G*_*g*_+*P*_*g*_	*E*_*g*_+*P*_*g*_

### 4.2 Game model analysis

(1) The benefits of patient recognition of treatment outcomes are:


$$\begin{array}{l} E_{x}=y\;*\;z\;*\;C_{t}+y\;*\;(1-z)\;*\;(C_{t}-C_{p}-C_{i})\\ \;+(1-y)\;*\;z\;*\;(C_{p}+C_{i}-C_{i})+(1-y)\;*\;(1-z)\;*\;C_{m}\; \end{array}$$

The benefits of patients not recognizing the outcome of the treatment were:


$$\begin{array}{l} E_{1-x}=y\;*\;z\;*\;(C_{t}+C_{p}+C_{i})+y\;*\;(1-z)\;*\;(C_{t}-C_{p})\\ \;+(1-y)\;*\;z\;*\;(C_{p}+C_{i}-C_{m})+(1-y)\;*\;(1-z)\;*\;(-C_{m}\\ \;-C_{p}-\;C_{i}) \end{array}$$

The average patient benefit was:


$$\begin{array}{l} \bar{E}=x{\;*\;E}_{x}+\left(1-x\right)\;*\;E_{1-x} \end{array}$$

The replication dynamic equation for the patient is:


$$\begin{array}{l} F(x)=x\;*\;(E_{x}-E)=x\;*\;[y\;*\;z\;*\;C_{t}+y\;*\;(1-z)\;*\;(C_{t}\\ \;-C_{p}-C_{i})+(1-y)\;*\;z\;*\;(C_{p}+C_{i}-C_{i})+(1-y)\;*\;(1\\ \;-z)\;*\;C_{m}-(x\;*\;E_{x}+(1-x)\;*\;E_{1-x}\;)]=x\;*\;(x-1)\\ \;*(2\;*\;C_{i}\;*\;y-2\;*\;C_{m}-C_{p}-C_{i}+2\;*\;C_{m}\;*\;y+C_{p}\;*\;y\\ \;+C_{i}\;*\;z+2\;*\;C_{m}\;*\;z+C_{p}\;*\;z-C_{i}\;*\;y\;*\;z-\;2\;*\;C_{m}\\ \;*y\;*\;z) \end{array}$$

(2) Benefits of reasonable treatment by medical institutions:


$$\begin{array}{l} E_{y}=x\;*\;z\;*\;(W_{t}+G_{s}+E_{r}-O_{c})+x\;*\;(1-z)\;*\;(W_{t}+G_{s}\\ \;+E_{r})+(1-x)\;*\;z\;*\;(W_{t}+G_{s}+E_{r}-O_{c})+(1-x)\;*\\ \;(1-z)\;*\;(W_{t}+G_{s}+E_{r}-\;O_{c}) \end{array}$$

Benefits of overmedication in medical institutions:


$$\begin{array}{l} E_{1-y}=x\;*\;z\;*\;(W_{m}-G_{f}-O_{c})+x\;*\;(1-z)\;*\;(W_{m}+G_{s}\\ \;-O_{c})+(1-x)\;*\;z\;*\;(W_{m}-G_{f}-O_{c})+(1-x)(1-z)\\ \;*(W_{m}-\;O_{c}) \end{array}$$

The average return for healthcare providers is:


$$\begin{array}{l} \bar{E}=y{*E}_{y}+\left(1-y\right)*E_{1-y} \end{array}$$

The replication dynamics equation for the medical institution is


$$\begin{array}{l} F(y)=y\;*\;(E_{y}-E)=y\;*\;[x\;*\;z\;*\;(W_{t}+G_{s}+E_{r}-O_{c})+x\;*\;(1\\ \;-z)\;*\;(W_{t}+G_{s}+E_{r})+(1-x)\;*\;z\;*\;(W_{t}+G_{s}+E_{r}-O_{c})\\ \;+(1-x)\;*\;(1-z)\;*\;(W_{t}+G_{s}+E_{r}-O_{c})-(y\;*\;E_{y}+(1\\ \;-y)\;*\;E_{1-y})]=y\;*\;(1-y)\;*\;(E_{r}+G_{s}-W_{m}+W_{t}\\ \;-G_{s}\;*\;x+G_{f}\;*\;z+O_{c}\;*\;x+G_{s}\;*\;x\;*\;z-\;O_{s}\;*\;x\;*\;z) \end{array}$$

(3) The benefits of strict government regulation:


$$\begin{array}{l} E_{z}=x*y*\left(E_{g}-C_{r}-G_{g}\right)+x\left(1-y\right)*\left(-C_{r}-G_{g}-P_{g}\right)\\ +\left(1-x\right)*y*\left(E_{g}-C_{r}-G_{g}+P_{g}\right)+\left(1-x\right)*\left(1-y\right)\\ {}*\left(P_{g}-C_{r}-G_{g}\right) \end{array}$$

Gains from lax government oversight:


$$\begin{array}{l} E_{1-z}=x*y*E_{g}+x*\left(1-y\right)*P_{g}+\left(1-x\right)*y*\left(E_{g}+P_{g}\right)\\ +\left(1-x\right)*\left(1-y\right)*\left(-E_{g}-P_{g}\right) \end{array}$$

The average benefit to government regulators is:


$$\begin{array}{l} \bar{E}=z*E_{z}+\left(1-z\right)*E_{1-z} \end{array}$$

The replication dynamic equation for the government regulator is:


$$\begin{array}{l} F(z)=z\;*\;[x\;*\;y\;*\;(E_{g}-C_{r}-G_{g})+x(1-y)\;*\;(-C_{r}-G_{g}-P_{g})\\ \;+(1-x)\;*\;y\;*\;(E_{g}-C_{r}-G_{g}+P_{g})+(1-x)\;*\;(1-y)\\ \;*\;(P_{g}-C_{r}-G_{g})\;-(z\;*\;E_{z}+(1-z)\;*\;E_{1-z})]=z\\ \;*(z-1)\;*\;(C_{r}-E_{g}+G_{g}-2\;*\;P_{g}+E_{g}\;*\;x+E_{g}\;*\;y+4\\ \;*\;P_{g}\;*\;x+2\;*\;P_{g}\;*\;y-E_{g}\;*\;x\;*\;y-\;4\;*\;P_{g}\;*\;x\;*\;y) \end{array}$$

### 4.3 Evolutionary system equilibrium analysis

The game process involving the government regulatory department, medical institutions, and patients is continuously evolving. Therefore, by establishing a tripartite game model replicator dynamic equation system, the equilibrium point of the tripartite game model can be calculated. The game process involving the government regulatory department, medical institutions, and patients is continuously evolving, which means that the probability of any strategy chosen by the three parties is time-dependent. According to the stability principle of differential equations, when all dynamic equations are equal to 0, it means that the entire dynamic system will tend to stabilize. Therefore, by establishing a tripartite game model replicator dynamic equation system and calculating the equilibrium point of the tripartite evolutionary game through *F*(*x*) = 0, *F*(*y*) = 0, *F*(*z*) = 0, we have:


$$\begin{array}{l} F(x)=x\;*\;(x-1)\;*\;(2\;*\;C_{i}\;*\;y-2\;*\;C_{m}-C_{p}-C_{i}+2\;*\;C_{m}\;*\;y\\ \;+C_{p}\;*\;y+C_{i}\;*\;z+2\;*\;C_{m}\;*\;z+C_{p}\;*\;z-C_{i}\;*\;y\;*\;z\\ \;-\;2\;*\;C_{m}\;*\;y\;*\;z)\\ F(y)=-y\;*\;(y-1)\;*\;(E_{r}+G_{s}-W_{m}+W_{t}-G_{s}\;*\;x+G_{f}\;*\;z\\ \;+O_{c}\;*\;x+G_{s}\;*\;x\;*\;z-\;O_{s}\;*\;x\;*\;z)\\ F(z)=z\;*\;(z-1)\;*\;(C_{r}-E_{g}+G_{g}-2\;*\;P_{g}+E_{g}\;*\;x+E_{g}\;*\;y\\ \;+4\;*\;P_{g}\;*\;x+2\;*\;P_{g}\;*\;y-E_{g}\;*\;x\;*\;y-\;4\;*\;P_{g}\;*\;x\;*\;y) \end{array}$$

According to Selton's research findings, in non-cooperative games, if the condition of information asymmetry holds, the evolutionarily stable strategy is a pure strategy. Therefore, it is only necessary to discuss the asymptotic stability of the eight local equilibrium points *E*1(0, 0, 0), *E*2(1, 0, 0), *E*3(0, 1, 0), *E*4(0, 0, 1), *E*5(1, 1, 0), *E*6(1, 0, 1), *E*7(0, 1, 1) and *E*8(1, 1, 1) that satisfy *F* = 0, *F*2 = 0, *F*3 = 0 in the above equations. According to the replicator dynamic equation of the three parties, the Jacobian matrix of the tripartite evolutionary game system can be obtained as follows:


$$\begin{array}{l} \;J=\left[\begin{array}{ccc} \frac{\partial F(x)}{\partial x} & \frac{\partial F(x)}{\partial y} & \frac{\partial F(x)}{\partial z}\\ \frac{\partial F(y)}{\partial x} & \frac{\partial F(y)}{\partial y} & \frac{\partial F(y)}{\partial z}\\ \frac{\partial F(z)}{\partial x} & \frac{\partial F(z)}{\partial y} & \frac{\partial F(z)}{\partial z} \end{array}\right]=\left[\begin{array}{ccc} J_{11} & J_{12} & J_{13}\\ J_{21} & J_{22} & J_{23}\\ J_{31} & J_{32} & J_{33} \end{array}\right]\;\\ J_{11}=(x-1)\;*\;(2\;*\;C_{i}\;*\;y-2\;*\;C_{m}-C_{p}-C_{i}+2\;*\;C_{m}\;*\;y\\ \;+C_{p}\;*\;y+C_{i}\;*\;z+2\;*\;C_{m}\;*\;z+C_{p}\;*\;z-C_{i}\;*\;y\;*\;z\\ \;-2\;*\;C_{m}\;*\;y\;*\;z)+x\;*\;(2\;*\;C_{i}\;*\;y-2\;*\;C_{m}-C_{p}-C_{i}+2\\ \;*\;C_{m}\;*\;y+C_{p}\;*\;y+C_{i}\;*\;z+2\;*\;C_{m}\;*\;z+C_{p}\;*\;z-C_{i}\;*\;y\\ \;*\;z-\;2\;*\;C_{m}\;*\;y\;*\;z)\\ J_{12}=x\;*\;(x-1)\;*\;(2\;*\;C_{i}+2\;*\;C_{m}+C_{p}-C_{i}\;*\;z-\;2\;*\;C_{m}\;*\;z\\ J_{13}=x\;*\;(x-1)\;*\;(C_{i}+2\;*\;C_{m}+C_{p}-C_{i}\;*\;y-\;2\;*\;C_{m}\;*\;y\\ J_{21}=y\;*\;(y-1)\;*\;\left(G_{S}-O_{C}-G_{i}\;*\;z+O_{c}\;*\;z\;\right)\\ J_{22}=-y\;*\;(E_{r}+G_{s}-W_{m}+W_{t}-G_{s}\;*\;x+G_{f}\;*\;z+O_{c}\;*\;x\\ \;+G_{s}\;*\;x\;*\;z-Oc\;*\;x\;*\;z)-(y-1)\;*\;(E_{r}+G_{s}-W_{m}+W_{t}\\ \;-G_{s}\;*\;x+G_{f}\;*\;z+O_{c}\;*\;x+G_{s}\;*\;x\;*\;z-\;O_{c}\;*\;x\;*\;z)\\ J_{23}=-y\;*\;(y-1)\;*\;(G_{f}+G_{s}\;*\;x-\;O_{c}\;*\;x)\\ J_{31}=z\;*\;(z-1)\;*\;(E_{g}+4\;*\;P_{g}-E_{g}\;*\;y-4\;*\;P_{g}\;*\;y),z\;*\;(z\\ \;-1)\;*\;(E_{g}+2\;*\;P_{g}-E_{g}\;*\;x-\;4\;*\;P_{g}\;*\;x)\\ J_{32}=z\;*\;(z-1)\;*\;(E_{g}+2\;*\;P_{g}-E_{g}\;*\;x-\;4\;*\;P_{g}\;*\;x)\\ J_{33}=z\;*\;(C_{r}-E_{g}+G_{g}-2\;*\;P_{g}+E_{g}\;*\;x+E_{g}\;*\;y+4\;*\;P_{g}\;*\;x\\ \;+2\;*\;P_{g}\;*\;y-E_{g}\;*\;x\;*\;y-4\;*\;P_{g}\;*\;x\;*\;y)+((z-1)\;*\;(C_{r})\\ \;-E_{g}+G_{g}-2\;*\;P_{g}+E_{g}\;*\;x+E_{g}\;*\;y+4\;*\;P_{g}\;*\;x+2\;*\;P_{g}\\ \;*\;y-E_{g}\;*\;x\;*\;y-\;4\;*\;P_{g}\;*\;x\;*\;y) \end{array}$$

According to the Lyapunov method, the stability of a differential system can be determined by the positive or negative values of the characteristic roots at the equilibrium points. When all the characteristic values (roots) of an equilibrium point are negative, the point is an evolutionarily stable strategy (asymptotically stable point). Substituting the eight pure strategy points into the Jacobian matrix in turn and obtaining the characteristic values of the equilibrium points, see Table 3.

**Table 3:** System equilibrium points and characteristic values.

**Balancing point**	**Jacobian matrix eigenvalues**			**Symbols**	**Deterministic conclusions**
	**λ1**	**λ2**	**λ3**		
*E*_1_(0, 0, 0)	*C*_*i*_+2 * *C*_*m*_+*C*_*p*_	*E*_*r*_+*G*_*s*_−*W*_*m*_+*W*_*t*_	*E*_*g*_−*C*_*r*_−*G*_*g*_+2 * *P*_*g*_	(+, *x, x*)	Instability point
*E*_2_(1, 0, 0)	−*C*_*i*_−2 * *C*_*m*_−*C*_*p*_	*E*_*r*_+*O*_*c*_−*W*_*m*_+*W*_*t*_	−*C*_*r*_−*G*_*g*_−2 * *P*_*g*_	(−, −, −)	ess
*E*_3_(0, 1, 0)	−*C*_*i*_	*W*_*m*_−*G*_*s*_−*E*_*r*_−*W*_*t*_	−*C*_*r*_−*G*_*g*_	(−, −, −)	ess
*E*_4_(0, 0, 1)	0	*E*_*r*_+*G*_*f*_+*G*_*s*_−*W*_*m*_+*W*_*t*_	*C*_*r*_−*E*_*g*_+*G*_*g*_−2 * *P*_*g*_	(0, +, *x*)	Uncertainty points
*E*_5_(1, 1, 0)	*C*_*i*_	*W*_*m*_−*O*_*c*_−*E*_*r*_−*W*_*t*_	−*C*_*r*_−*G*_*g*_	(+, *x*, −)	Instability point
*E*_6_(1, 0, 1)	0	*E*_*r*_+*G*_*f*_+*G*_*s*_−*W*_*m*_+*W*_*t*_	*C*_*r*_+*G*_*g*_+2 * *P*_*g*_	(0, *x*, +)	Uncertainty points
*E*_7_(0, 1, 1)	−*C*_*i*_−*C*_*p*_	*W*_*m*_−*G*_*f*_−*G*_*s*_−*E*_*r*_−*W*_*t*_	*C*_*r*_+*G*_*g*_	(−, *x*, +)	Instability point
*E*_8_(1, 1, 1)	*C*_*i*_+*C*_*p*_	*W*_*m*_−*G*_*f*_−*G*_*s*_−*E*_*r*_−*W*_*t*_	*C*_*r*_+*G*_*g*_	(+, *x*, +)	Instability point

x indicates unknown symbol.

From Table 3, it can be seen that there may be two evolutionary stable equilibrium points in the evolutionary game system: when *E*_2_(1, 0, 0) becomes the equilibrium point, the stability condition *E*_*r*_+*O*_*c*_+*W*_*t*_ < *W*_*m*_, needs to be met. When *E*_3_(0, 1, 0) becomes the equilibrium point, Stability condition *G*_*s*_+*W*_*t*_+*E*_*r*_ < *W*_*m*_ needs to be satisfied?

While this study primarily focuses on analyzing pure strategy equilibria for simplicity and tractability, it is important to acknowledge that mixed strategy equilibria can also exist in evolutionary game dynamics, particularly under conditions where no pure strategy satisfies the stability conditions or when strategy payoffs are closely balanced. According to the replicator dynamic framework, mixed strategies may emerge as internal equilibria where all strategies coexist with non-zero probabilities. However, in our numerical simulations, no stable interior fixed points (i.e., mixed-strategy equilibria) were observed under the given parameter settings. Future research could further explore these possibilities using alternative dynamic models (e.g., best-response dynamics or stochastic perturbations).

## 5 Numerical simulation analysis

We utilized MATLAB R2020b's simulation toolkit to perform multiple runs, each with different initial conditions, to observe the evolution of strategies over time. Specifically, we used MATLAB's built-in ODE45 differential equation solver to numerically integrate the system of replicator dynamic equations derived in Section 4.2. The simulation time horizon was set to 50 iterations (or time units), and the time step was adjusted adaptively by the solver to balance accuracy and efficiency. Each simulation run was initialized with different probability values for the strategy vectors of patients (x), medical institutions (y), and government regulators (z), typically starting from 0.5 to reflect neutral initial attitudes. The output of each simulation included the trajectories of strategy proportions over time, which were then visualized using phase diagrams and time-series plots. These visualizations allowed us to analyze the convergence behavior, assess the stability of equilibrium points, and identify critical parameter thresholds influencing strategic outcomes. The analysis focused on how changes in parameters such as fiscal subsidies (*G*_*s*_), government fines (*G*_*f*_), and other key variables influenced the stakeholders' decisions. For instance, we varied the government fines (*G*_*f*_) from minimal to punitive levels to observe its impact on healthcare institutions' propensity toward overtreatment. The outcomes were evaluated based on the convergence of strategies toward equilibrium, indicating a stable state where no player has an incentive to deviate from their chosen strategy. This process allowed us to draw conclusions about the effectiveness of various policy measures in reducing excessive medical care.

### 5.1 Evolutionary stabilization strategy

The data for variables such as “hospital healthcare operating costs,” “government financial subsidies,” “government fines,” and “regulatory costs” were specifically obtained from authoritative sources. According to the “China Health and Wellness Statistical Yearbook 2022” (63), the total national health expenditure for 2022 was 8,484.67 billion yuan. This included government financial subsidies amounting to 2,391.64 billion yuan, social health expenditure of 3,801.58 billion yuan, and personal health expenditure of 2,291.45 billion yuan. Additionally, the “2022 healthcare institutions Security Business Development Statistical Bulletin” (64) reported that the healthcare institutions Security Administration penalized 12,029 medical institutions, recovering a total of 18.84 billion yuan in medical insurance funds, imposing fines totaling 13.866 billion yuan, and collecting 1.893 billion yuan in liquidated damages.

To ensure our simulation reflects a scaled representation of the actual economic context within the Chinese healthcare system, we set our parameters proportionally based on these official statistics. For instance, we assume the following initial strategies and parameters: *C*_*m*_ (Cost of overmedicalization) = 6, *C*_*t*_ (Benefits of appropriate diagnosis and treatment) = 2, *C*_*p*_(Probability of patient complaints) = 10, *C*_*i*_ (Compensation income) = 0.5, *W*_*m*_ (Income from overmedicalization) = 9, *W*_*t*_(Income from appropriate diagnosis and treatment) = 4, *G*_*f*_(Government fines) = 6, *G*_*s*_(Government financial subsidies) = 5, *E*_*r*_ (Benefits from good reputation) = 2, *C*_*r*_ (Regulatory costs) = 1, *O*_*c*_ (Operational costs of hospital medical services) = 1.5, *E*_*g*_ (Social benefits) = 7, *G*_*g*_ (Remediation costs) = 2, *P*_*g*_ (Government credibility) = 1. For the equilibrium point *E*_2_(1, 0, 0) to be valid, the condition *E*_*r*_+*O*_*c*_+*W*_*t*_ < *W*_*m*_ must be satisfied. Therefore, by setting these initial parameters and assuming the initial strategies for x, y, and z are x = 0.5, y = 0.5, and z = 0.5, respectively, we ensure that our model accurately reflects the dynamics within the healthcare system. These values were selected to provide a realistic basis for our simulation, reflecting both empirical data and theoretical considerations. By integrating the data sources and parameter assumptions in this manner, we aim to provide a clear and coherent foundation for our evolutionary game analysis.

**Figure 1:**
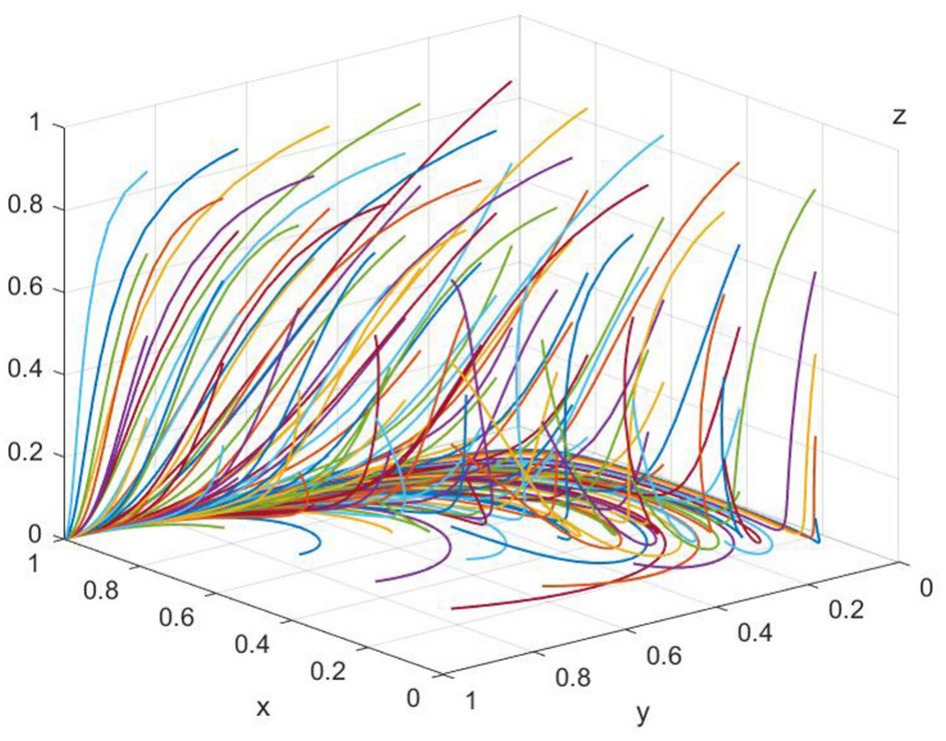
Simulation of equilibrium point (1,0,0) parameters (evolved 50 times). The x-axis represents the proportion of patients recognizing treatment outcomes, the y-axis denotes the proportion of healthcare institutions providing reasonable treatment, and the z-axis refers to the proportion of government regulators adopting strict supervision. Different colored trajectories represent different initial probability settings, all converging toward the equilibrium under the baseline scenario. The figure visually demonstrates the dynamic stability and convergence tendency of the tripartite system.

As illustrated in Figure 1, when the cost of overtreatment (*W*_*m*_) incurred by patients exceeds the sum of the benefits from a good reputation for government regulatory departments (*E*_*r*_), operational costs of hospital medical services (*O*_*c*_), and income from reasonable diagnosis and treatment by medical institutions (*W*_*t*_), the ultimate evolutionary outcome will always be (1, 0, 0). That is, the strategy choices for patients, medical institutions, and government regulatory departments will converge on “non-acknowledgment of treatment results,” “excessive treatment,” and “lenient regulation,” respectively. The curves in the figure clearly demonstrate the system's convergence toward this point.

For the equilibrium point *E*_3_(0, 1, 0), the condition *G*_*s*_+*W*_*t*_+*E*_*r*_ < *W*_*m*_ needs to be satisfied. Similarly, assume that the initial strategies for x, y and z are x = 0.5, y = 0.5 and z = 0.5, respectively. To satisfy the above condition, assume that *C*_*m*_ = 6, *C*_*t*_ = 2, *C*_*p*_ = 10, *C*_*i*_ = 0.5, *W*_*m*_ = 9, *W*_*t*_ = 7, *G*_*f*_ = 6, *G*_*s*_ = 5, *E*_*r*_ = 2, *C*_*r*_ = 1, *O*_*c*_ = 1.5, *E*_*g*_ = 7, *G*_*g*_ = 2, *P*_*g*_ = 1.

**Figure 2:**
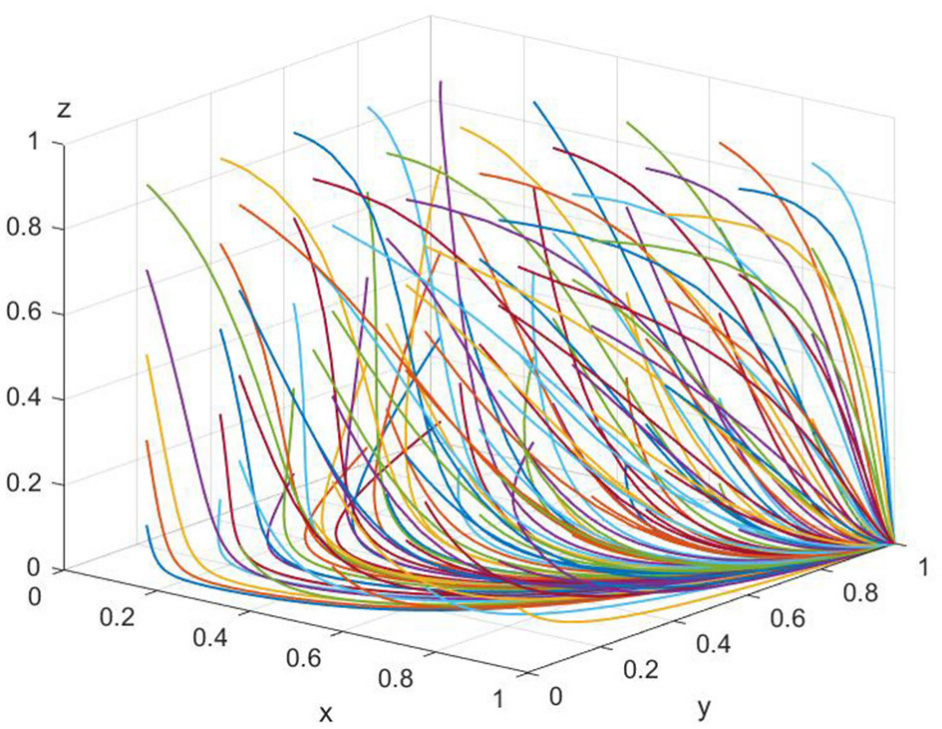
Simulation of equilibrium point (0,1,0) parameters (50 times of evolution). The axes are defined as in Figure 1. Each colored line indicates a unique combination of initial strategy probabilities for the three stakeholders. This figure illustrates how varying initial attitudes still lead to the same or similar equilibrium points, highlighting the model's robustness to initial conditions.

As shown in Figure 2, when the overtreatment income (*W*_*m*_) of medical institutions is higher than the sum of the government's fiscal subsidies (*G*_*s*_), the reasonable medical treatment income (*W*_*t*_) of the medical institutions, and the good reputation revenue (*E*_*r*_) of the government regulatory department, the equilibrium point gradually shifts from point (1, 0, 0) to point (0, 1, 0) as the evolution progresses. At this time, the optimal strategies for the parties involved in the game are “patients acknowledging the treatment outcome,” “medical institutions providing reasonable treatment,” and “government regulatory departments exercising lenient supervision.” The figure highlights how the strategies evolve and stabilize at this equilibrium point under the given conditions.

In the paper, Figures 1, 2 are presented as part of the sensitivity analysis conducted through evolutionary game theory simulations. Figure 1 corresponds to the equilibrium point (1,0,0), with each color of the lines representing a different parameter that affects the stability of this equilibrium. The variety of colors is used to differentiate the impact of each parameter, such as hospital operating costs or government financial subsidies, on the system's dynamics. Here, each color is specifically chosen to indicate a unique variable and its effect on the equilibrium under investigation. Through these figures, we aim to convey a clear and intuitive understanding of the parameters that are critical to the system's balance and how their modulation can guide the development of collaborative governance policies to counteract excessive medical care. The use of distinct colors across the lines in both figures allows for an immediate visual distinction between the different parameters being analyzed, providing a more accessible way to comprehend the complex interactions at play.

### 5.2 Parameter sensitivity analysis

To validate the influence of parameter settings on the evolution paths and strategic stability of the model, this section conducts a comprehensive sensitivity analysis. The analysis covers key variables including government subsidies, fines, regulatory costs, remediation costs, reasonable treatment income, and overtreatment income. In addition, for variables that are difficult to measure monetarily—such as Social Benefits (*E*_*g*_), Government Credibility (*P*_*g*_), and Patient Complaint Probability—we adopted a standardized scale of [1–10] and simulated across different value intervals to test their impact on the evolution of strategies and equilibrium outcomes.

Simulation results show that while different values slightly affect the convergence speed, the final evolutionary equilibrium remains structurally stable. This demonstrates the robustness of the model and confirms that the simulation conclusions are not overly sensitive to specific parameter choices. The results thereby enhance the overall reliability and credibility of the model.

In the context of China's healthcare reform, the system's stable equilibrium point *E*_3_(0, 1, 0) represents the optimal strategic choice at present. At equilibrium point *E*_3_(0, 1, 0), the strategy combination of the three parties is “patients acknowledging treatment outcomes,” “medical institutions providing reasonable treatment,” and “government exercising lenient supervision.” Overtreatment by medical institutions could harm the interests of patients within the same system and reduce their trust in the government regulatory departments. Only by medical institutions undertaking social responsibility and implementing reasonable treatment can the patients' treatment costs be effectively reduced, trust in the healthcare system be enhanced, and government resources not be wasted on strong supervision of the medical institutions' treatment behavior when they are already choosing reasonable treatment strategies. Therefore, the government can appropriately reduce regulation. Meanwhile, under certain conditions, the equilibrium points (0, 1, 0) and (1, 0, 0) may be mutually convertible. When the game system is at equilibrium point (1, 0, 0), it is unfavorable for improving overall social welfare. Thus, this study conducts sensitivity analysis on some key parameters among the three potential equilibrium points to better reveal the important influencing factors of each party's strategic choice. These key parameters include fiscal subsidies for government medical institutions (*G*_*s*_), reasonable treatment income (*W*_*t*_), overtreatment income (*W*_*m*_), government fines (*P*_*g*_), government rectification costs (*G*_*g*_), and government supervision costs (*C*_*r*_).

Delving deeper into the sensitivity analysis, we meticulously examined how specific variations in government policies (e.g., increases in fiscal subsidies or fines) directly impact the strategic choices of healthcare institutions and the regulatory stance of government departments. The choice of a 10% increase in government fines is based on practical considerations derived from recent enforcement patterns in China's healthcare regulation. For instance, according to the 2022 Healthcare Security Administration Bulletin, year-over-year adjustments in fines and recovered funds for overtreatment violations have ranged between 8 and 15% in several provinces. Therefore, a 10% increment was selected as a representative and conservative scenario for simulation purposes. Preliminary tests with 5%, 10%, and 15% increments showed consistent directional effects, with differences only in convergence speed. By presenting detailed simulations of these scenarios, we offer concrete evidence on the potential efficacy of various policy measures. For instance, we discovered that a 10% increase in government fines for overtreatment significantly deters healthcare institutions from adopting overtreatment strategies, highlighting the critical role of punitive measures in curbing excessive medical care.

**Figure 3:**
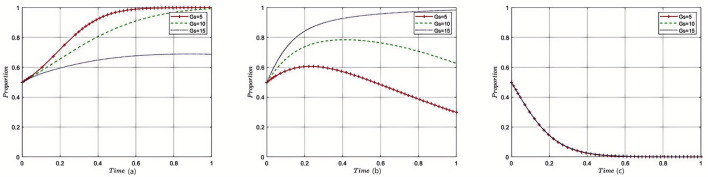
Sensitivity analysis of government subsidies. Each subplot traces the time evolution of strategy proportions for the three participant groups. The distinct lines indicate outcomes under varying levels of government subsidies. Results show that increased subsidies incentivize reasonable medical practice by institutions and accelerate convergence toward cooperative equilibria.

**Figure 4:**
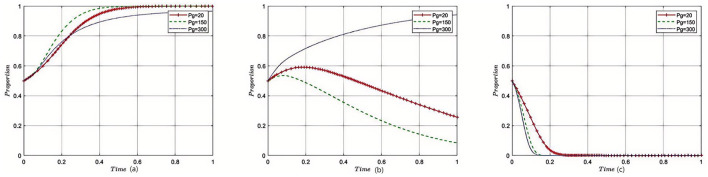
Sensitivity analysis of government fines on medical institutions. Each curve displays how strategy adoption among stakeholders shifts over time in response to changing penalty strength. The figure illustrates that enhanced penalties discourage overtreatment and encourage compliance, while also affecting the government's supervisory intensity.

**Figure 5:**
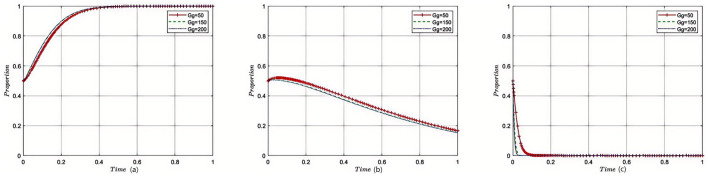
Sensitivity analysis of government remediation costs. The subplots reveal how changes in rectification costs alter the pace and direction of strategy evolution among the three groups. Higher rectification costs strengthen internal and external constraints, reducing overtreatment tendencies in healthcare institutions.

**Figure 6:**
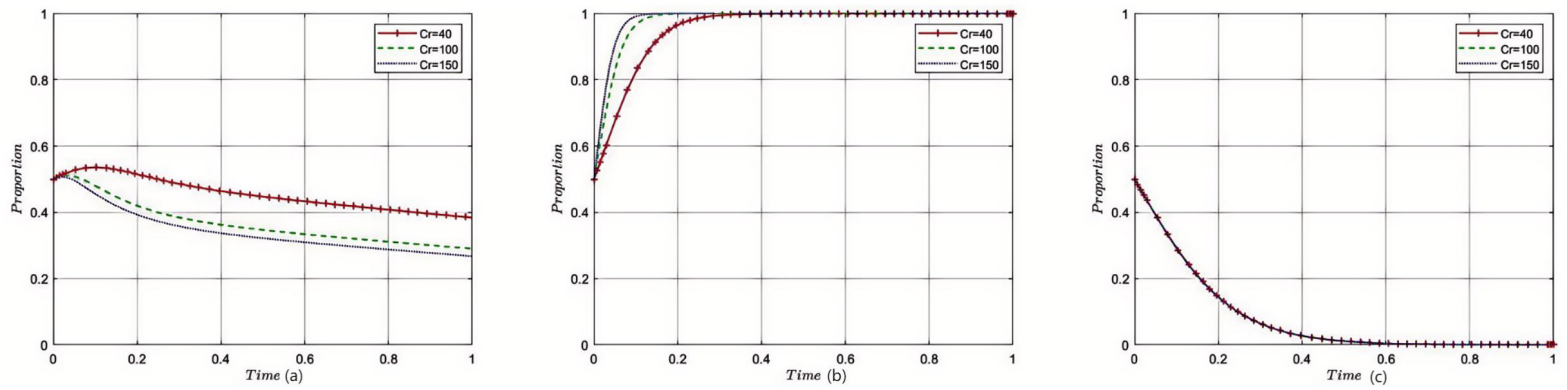
Sensitivity analysis of government regulatory costs. The plots show how changes in income from providing reasonable care affect strategic evolution over time. Greater rewards for reasonable treatment motivate providers to prioritize patient-centered care, with little effect on regulator behavior.

**Figure 7:**
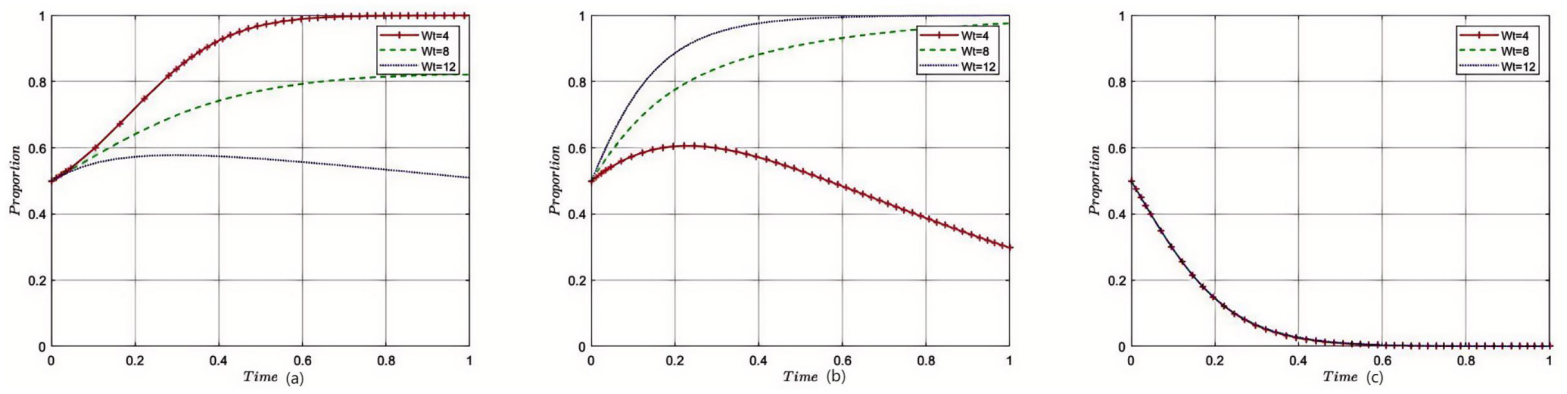
Sensitivity analysis of reasonable treatment revenue of medical institutions. These curves demonstrate that as the incentives for overtreatment increase, providers become more likely to pursue such strategies, while patient approval declines. The figure underscores the importance of aligning provider incentives with rational medical practice.

**Figure 8:**
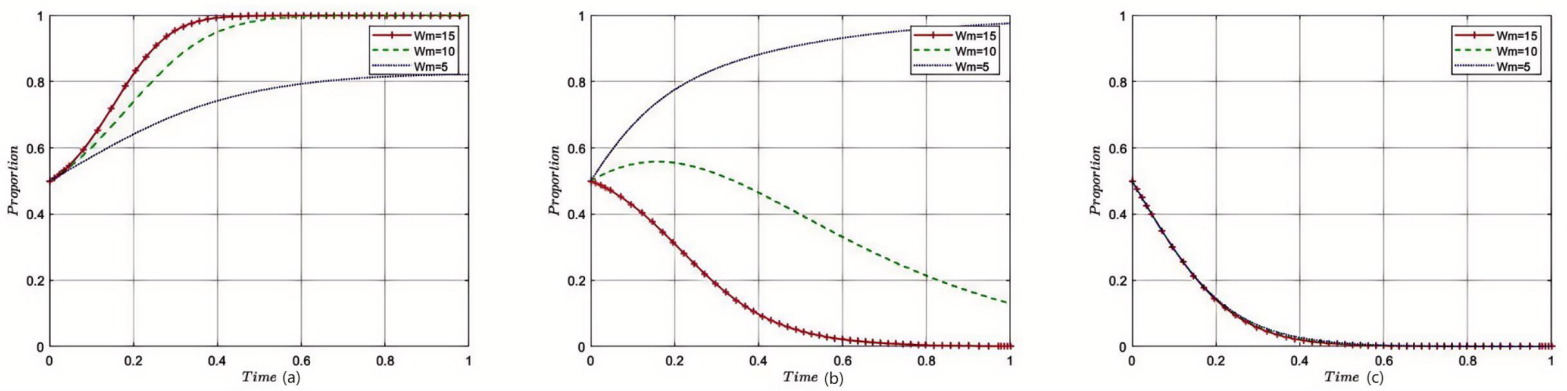
Sensitivity analysis of excessive medical care in medical institutions. The graphs show the effect of varying supervisory costs on the speed and final distribution of stakeholder strategies. When supervision is more costly, regulatory efforts decrease, leading to higher risk of overtreatment by healthcare institutions.

Figures 3–8 in the paper display phase diagrams that capture the interaction dynamics among patients, medical institutions, and government regulatory bodies within our evolutionary game theory framework. These diagrams illustrate how each party's strategies evolve over time, given the continuous feedback between the healthcare system's entities. The lines chart the strategic trajectory of each stakeholder group, influenced by their own objectives and the actions of the others. For instance, they might show shifts toward more cost-effective treatment options by patients, ethical practice adoption by medical institutions, or varying intensities of oversight by government regulators. By focusing on different equilibrium states, the diagrams reveal the stability of these strategies and the potential for policy interventions to shape the system toward optimal outcomes. This visual analysis aids in understanding the delicate balance required for collaborative governance and the conditions that will either support or undermine the reduction of excessive medical care, thereby providing a strategic roadmap for policy formulation.

#### 5.2.1 The impact of government financial subsidies to medical institutions on the tripartite evolutionary game

Under the equilibrium conditions satisfying point *E*_2_(0, 1, 0) assume *G*_*s*_ = 5, 10, 15; the simulation evolution in this case is illustrated in Figure 3. The results indicate that as the amount of government fiscal subsidies to medical institutions increases, the patients' choice of medical treatment strategy is affected, but there is no strategic impact on the government itself. At this point, increasing the fiscal subsidies to medical institutions by the government can reduce overtreatment behaviors and enhance the enthusiasm of medical institutions for providing reasonable treatment, resulting in good reputation revenue for the medical institutions. For the government, reasonable treatment provided by medical institutions can reduce supervision and rectification costs. The amount of fiscal subsidies provided to medical institutions does not affect the decision-making of the government regulatory departments.

#### 5.2.2 The impact of government fines on the tripartite evolutionary game

Under the equilibrium conditions satisfying point *E*_2_(0, 1, 0), assume *P*_*g*_ = 20, 150, 300, the simulation evolution in this case is illustrated in Figure 4. The results demonstrate that the higher the fines imposed by the government on medical institutions, the more likely patients are to disapprove of overtreatment governance. When the fines on medical institutions are relatively low, medical institutions tend to provide reasonable treatment. However, when the government's fines exceed a certain threshold, the government will relax supervision, leading to a rapid increase in the probability of overtreatment by medical institutions. The higher the fines imposed by the government regulatory departments on medical institutions, the more likely they are to adopt lenient supervision. The impact of the amount of fines on the decision-making of government regulatory departments is relatively small, and when the amount of fines reaches a certain threshold, it converges toward strict supervision.

#### 5.2.3 Impact of government remediation costs on the evolutionary game of the three parties

Under the equilibrium conditions satisfying point *E*_2_(0, 1, 0), assume *G*_*g*_ = 50, 150, 200, the simulation evolution in this case is illustrated in Figure 5. The results show that with the increase in government political costs, medical institutions will also choose reasonable strategies, the constraints will be strengthened, and the probability of overtreatment will be reduced, having almost no impact on patients' medical treatment decisions. The level of rectification costs has little influence on the government regulatory departments, and it will eventually converge toward reduced strict supervision.

#### 5.2.4 Impact of government regulatory costs on the tripartite evolutionary game

Under the equilibrium conditions satisfying point *E*_2_(0, 1, 0), assume *C*_*r*_ = 40, 100, 150, the simulation evolution in this case is illustrated in Figure 6. The results show that when the supervision costs of government regulatory departments are very low, medical institutions will be subject to more supervision, and thus, they are more inclined to provide reasonable treatment. However, as supervision costs increase, government regulatory departments reduce the intensity of supervision on medical institutions, making them more prone to overtreatment. The level of supervision costs has almost no impact on the decision-making of government regulatory departments.

#### 5.2.5 Impact of reasonable treatment income on the tripartite evolutionary game

Under the equilibrium conditions satisfying point *E*_2_(0, 1, 0), assume *W*_*t*_ = 4, 8, 12; the simulation evolution in this case is illustrated in Figure 7. The results show that the higher the income from reasonable treatment provided by medical institutions, the lower the likelihood of patients approving overtreatment outcomes, and the more medical institutions tend to provide reasonable treatment. The level of reasonable treatment income has almost no impact on the decision-making of government regulatory departments. For medical institutions, the higher the income from reasonable treatment, the higher the fiscal revenue, and the more subsidies they will receive; for the government, as medical institutions adhere to reasonable treatment, the subsidies provided by the government to medical institutions will also increase accordingly; for patients, as the cost of reasonable medical treatment increases, their enthusiasm for receiving treatment from medical institutions will decrease.

#### 5.2.6 Impact of excessive medical income on the tripartite evolutionary game

Under the equilibrium conditions satisfying point *E*_2_(0, 1, 0), assume *W*_*m*_ = 15, 10, 5; the simulation evolution in this case is illustrated in Figure 8. The results show that the higher the income from overtreatment by medical institutions, the more likely patients are to disapprove of overtreatment. An increase in overtreatment income will reduce the willingness of medical institutions to manage, and the higher the overtreatment income, the more medical institutions tend to avoid managing. When overtreatment income exceeds a threshold, medical institutions will change their strategies and eventually converge to non-management. Changes in overtreatment income have no impact on government regulatory departments. At this point, the benefits of overtreatment for medical institutions gradually decrease, and the income of medical institutions becomes increasingly lower; for the government, as medical institutions engage in overtreatment, the government's regulation of medical institutions will also relax accordingly; for patients, as the cost of overtreatment becomes lower, their enthusiasm for receiving treatment from medical institutions will increase.

## 6 Results and recommendations

### 6.1 Study results

In this study, from the perspective of bounded rationality, we employ the evolutionary game theory to establish an evolutionary game model for the collaborative governance of excessive medical treatment among government regulatory departments, medical institutions, and patients. The model investigates the evolutionary strategy equilibrium of different stakeholders and the impact of various factors on the strategic evolution of the three parties. We analyze the intrinsic logic of government regulatory departments in promoting reasonable medical treatment measures by implementing economic incentives or penalties for unreasonable medical treatments in medical institutions. This study also explores the inherent conditions for achieving collaborative governance by taking advantage of the leading role of government regulatory departments. Within this theoretical framework, further empirical testing of the theoretical results with real-world data is the next direction for research. The conclusions of this study are as follows:

In interpreting the results of our evolutionary game theoretical model, it is evident that the strategic interactions between government regulatory departments, medical institutions, and patients are highly sensitive to fiscal dynamics such as subsidies, fines, and operational costs. In interpreting the results of our evolutionary game theoretical model, it is evident that the strategic interactions between government regulatory departments, medical institutions, and patients are highly sensitive to fiscal dynamics such as subsidies, fines, and operational costs. While subsidies for reasonable treatments may reduce some over-treatment practices, defining and monitoring “reasonable treatment” presents a significant challenge. Without clear and standardized definitions, the risk of misuse remains. Additionally, subsidies alone may not fully eliminate over-treatment, particularly if the financial incentives for over-treatment exceed the subsidies provided. Therefore, an integrated approach that combines financial incentives with stronger regulatory oversight, technological tools such as AI for monitoring, and penalties for over-treatment may be necessary to ensure the long-term effectiveness of such policies.

This study's simulations suggest that increasing government subsidies and income for reasonable treatments effectively curbs overmedicalization, echoing Liu et al. (6, 7) findings on government incentives boosting reasonable medical services. Differently, this work delves into how these incentives impact the stability of strategy choices among involved parties, offering policymakers theoretical model-based evidence. Moreover, it highlights patient trust's crucial role in enhancing reasonable medical service provision, thus broadening the understanding of patient influence in the literature.

The behavior choices of government regulatory departments, medical institutions, and patients mutually influence each other. However, patients' decisions are constrained by information asymmetry, making it difficult for them to identify instances of overtreatment. Their decisions largely depend on trust in medical professionals, which limits their direct influence on the strategies of hospitals and government regulators. Despite this, patients still play a role in shaping the feedback mechanisms within the system, particularly through their reactions to treatment outcomes. Conversely, engagement in excessive medical treatment by institutions leads to vigorous patient disapproval and stricter government supervision. This dynamic is supported by the findings of Beaussier et al. (65) who highlight the complex relationship between regulatory practices and healthcare quality, particularly in the context of the NHS. Their study underlines how flexible and risk-based policy instruments can impact the effectiveness and legitimacy of healthcare regulation, paralleling our observations on government regulatory strategies.

Additionally, the degree of patient recognition influences government decision-making, affecting the amount of financial subsidies provided to medical institutions. These institutions respond to economic incentives or penalties from the government, as noted by Karanicolas et al. (66). Their research emphasizes the importance of methodological approaches in understanding the effects of healthcare policies, resonating with our findings on the sensitivity of medical institutions to economic incentives. However, they also caution about the potential for high economic incentives to impose undue fiscal pressure on governments, mirroring our observations on the balance required in policy implementation.

Medical institutions are critical to the “source” control of excessive medical treatment. Reducing excessive medical treatment behavior in medical institutions can be achieved by increasing government financial subsidies, enhancing the income of appropriate treatments, and strengthening supervision and rectification efforts.

Compared to traditional analytical methods in healthcare governance—such as static optimization models—the evolutionary game theory approach offers a dynamic perspective that captures the ongoing strategic adaptations of stakeholders. While static models provide a snapshot of system behavior under a set of assumptions, they often fail to account for the iterative learning and feedback mechanisms inherent in real-world policy environments. In contrast, our simulation results resonate with empirical observations from the Chinese healthcare system, offering a more nuanced understanding of the behavior underpinning excessive medical care. Moreover, the evolutionary approach aligns with the emerging body of research employing complex adaptive systems theory to understand healthcare dynamics, reflecting a broader shift in the academic discourse toward models that embrace the intricacies of policy ecosystems.

The results of the evolutionary game model suggest that stricter regulatory measures, including increased government fines and improved enforcement transparency, are necessary to shift healthcare institutions' behavior toward reasonable treatment strategies. The model demonstrates that when penalties for overtreatment are too low or when oversight is lax, medical institutions tend to prioritize profit over patient welfare, leading to continued overtreatment behaviors. Therefore, policy recommendations, such as raising fines and increasing the transparency of government oversight, are directly supported by the model's predictions of strategic behavior under various parameter settings.

### 6.2 Suggestions for countermeasures

As mentioned earlier, the reasonable regulation of excessive medical treatment should consider the roles played by various stakeholders in the phenomenon and propose targeted and feasible regulatory methods and suggestions based on their characteristics and shortcomings. In this context, it is pertinent to also compare fiscal subsidies and government fines, our primary focus, with other regulatory strategies such as direct regulation, accreditation programs, public reporting, and market-based incentives. This comparison will evaluate their relative effectiveness, feasibility, and impacts on healthcare outcomes, thus offering a more comprehensive perspective on policy options for mitigating medical overuse. The author will now provide rationalized suggestions for the reasonable regulation of excessive medical treatment from different perspectives, integrating these comparative insights.

#### 6.2.1 Strengthen punishment measures and legal enforcement mechanisms for excessive medical treatment

While the existing legal framework in China, including the “Civil Code,” “Physician Law,” and “Regulations on the Prevention and Treatment of Medical Disputes,” provide foundational legal constraints, the enforcement of these laws remains insufficient to curb the issue of excessive medical treatment. The evolutionary game model analysis shows that without stringent penalties (represented by the government fines in the model), medical institutions have little incentive to adhere to reasonable treatment practices, as the benefits from overtreatment (*G*_*f*_) often outweigh the costs. Therefore, increasing fines and implementing more rigorous enforcement of existing laws are necessary to shift the equilibrium toward strategies that discourage overtreatment. Furthermore, transparency in the enforcement process, as well as public reporting on regulatory actions, can increase patient trust and indirectly pressure medical institutions to comply with reasonable practices, as modeled by the sensitivity of patient trust in the evolutionary game.”

In Germany, the orderly development of the medical and health industry is inseparable from legal constraints, and the government achieves the effectiveness of medical and health system reform through legislation. In China, the relevant law on excessive medical treatment is the “Tort Liability Law,” which, for the first time, incorporates excessive examinations in excessive medical treatment into the legal category. The promulgation of this legislation is progressive for the legal regulation of excessive medical treatment. However, the “Tort Liability Law” only stipulates excessive examination behaviors in excessive medical treatment without mentioning excessive medication, excessive surgery, or excessive healthcare. These improper medical behaviors can also infringe upon patients' legitimate rights and interests, causing economic losses and psychological burdens to patients. Therefore, limiting the scope of regulating excessive medical treatment to excessive examinations in the “Tort Liability Law” appears one-sided in protecting patients' legitimate rights and interests and needs further expansion and interpretation.

Based on the sensitivity analysis results (see Figure 4), our study finds that once government fines for overtreatment exceed a certain threshold, healthcare institutions are significantly more likely to shift toward appropriate treatment strategies, and the system evolves toward a stable state characterized by rational care. Therefore, it is advisable to establish a minimum fine threshold—for instance, no less than the median gap between revenues from overtreatment and those from appropriate treatment. This ensures that economic penalties remain an effective deterrent. Furthermore, policymakers should avoid excessively high fines, which may lead to reduced regulatory efforts by government departments. Thus, striking a balance between “deterrence” and “regulatory sustainability” is critical in the design of legal enforcement mechanisms.

#### 6.2.2 Improving the healthcare regulatory system and enhancing government regulatory functions

The government can implement a “separation of management and supervision” in healthcare institutions through delegation, where a supervisory committee is established to regulate the behavior of these institutions continuously. The third-party organization entrusted with this responsibility is not subject to any department, institution, or organization. It supports preventing, detecting, and investigating potential cases of excessive medical treatment. At the same time, the government should address the issues of excessive medical treatment and the supervision of such practices and broaden channels for public supervision. Establishing a public participation and supervision mechanism that includes patients, media, and various industries, incorporating government cooperation, self-discipline within the healthcare industry, and multi-channel social supervision will effectively curb the problem of inadequate regulation of excessive medical treatment.

Harnessing the self-disciplinary functions of industry associations is crucial. The physician association exercises the authority to handle physician licenses. Suppose a physician is found to have violated professional rules. In that case, the association has the right to revoke their license and, in severe cases, prohibit them from re-entering the medical profession. The physician association formulates “Clinical Diagnosis and Treatment Guidelines,” which include diagnostic and treatment standards and medication protocols for various diseases. Physicians must strictly adhere to these standards during their practice. If deviations from the guidelines are found, or if a physician violates them for personal gain, the physician association has the authority to review the case and impose penalties such as fines, license revocation, and market exclusion as punitive measures.

To enhance the government's ability to detect excessive medical treatment, it is recommended to adopt more advanced technological solutions, such as big data analytics and AI, which can analyze large volumes of healthcare data and identify patterns of overtreatment. Additionally, increasing the transparency of hospital treatment records and encouraging patient participation through complaint mechanisms can further aid in the detection process. These measures, combined with existing audits and inspections, will help mitigate the impact of information asymmetry and improve the overall effectiveness of government regulation.

#### 6.2.3 Improving the healthcare insurance payment system

Currently, various regions in China are actively exploring payment reform methods, such as payment by disease category or payment by Diagnosis-Related Groups (DRGs). Although some places have already implemented payment based on disease category scores, where the payment prices for diseases are lowered through competition between hospitals, the effectiveness of treatment has not been fully considered. If effectiveness and safety cannot be guaranteed, such payment reform policies fail to achieve their original intent and end up rewarding low-cost healthcare institutions and doctors rather than high-value ones.

China should establish an incentive-compatible payment system to guide the optimal allocation of healthcare resources. By guiding payment methods, it can standardize the diagnostic and treatment behaviors of healthcare providers and improve the efficiency of healthcare resource allocation. These measures include implementing per capita payment for general practitioners, implementing per diem or per disease category payment for hospitals, and implementing payment by Diagnosis-Related Groups (DRGs) for inpatient services.

#### 6.2.4 Strengthening internal constraint mechanisms in healthcare institutions

Enhancing internal institutional development and strengthening constraint mechanisms are crucial in healthcare institutions. Enhancing internal institutional development and strengthening constraint mechanisms are crucial in healthcare institutions. While increasing government subsidies and income for reasonable treatments is an important step, it may not fully eliminate over-treatment, particularly if the financial benefits of over-treatment remain higher than the subsidies. To address this, hospitals should bolster internal institutional development by establishing a scientific medical performance evaluation mechanism and an internal distribution incentive mechanism.

The pursuit of profit is a key driver of overtreatment behaviors in healthcare institutions. This aligns with the evolutionary game model's assumption that healthcare providers are motivated by financial incentives, and without adequate penalties or oversight, overtreatment becomes a dominant strategy. To address this issue, it is crucial to strengthen internal accountability mechanisms within healthcare institutions, such as performance evaluations that prioritize patient outcomes over financial performance. Moreover, empowering professional ethics committees and strengthening peer review processes within institutions can help mitigate the influence of profit-driven motives.

They are implementing clinical pathway management to improve healthcare quality. Clinical pathways are standardized methods of diagnosis and treatment. The examination, laboratory tests, consultations, treatments, surgeries, postoperative recovery, hospital stay duration, and patient costs can be standardized and controlled by formulating clinical treatment pathways. Clinical pathways enhance healthcare quality, reduce medical expenses, shorten hospital stays, control excessive medical treatment, and strengthen doctor-patient communication. They represent a management model for continuous improvement in healthcare quality.

Additionally, as shown in Figure 3, the simulation results indicate that when government financial subsidies to healthcare institutions reach a specific threshold (e.g., Gs ≥ 5 in our model), medical institutions are incentivized to adopt appropriate treatment strategies, leading to a desirable equilibrium state. Accordingly, it is recommended that government departments define a minimum subsidy threshold based on regional medical service costs and insurance fund capacity. This threshold should be treated as a foundational economic lever to promote rational care. Furthermore, dynamic adjustments to this threshold—guided by continuous simulations and calibration with real-world data—can ensure optimal use of fiscal resources and enhance the efficacy of internal hospital governance mechanisms.

#### 6.2.5 Cultivating patient awareness of rights and enhancing subjective value perception in governance participation

With the rapid advancement of social intelligence, the public's cultural level and learning capabilities are continuously improving, and their analytical judgment will play an increasingly significant role in the governance of social affairs. “Psychological empowerment,” which refers to the cultivation of awareness of rights, is the first step in empowering insured individuals. Insured individuals must gradually enhance their subjective value perception in governance participation, clearly understanding the need to safeguard their health rights and economic interests. Insured individuals and the government have overlooked the potential for creating cooperative benefits in the current healthcare service market due to the inertia of rights awareness.

### 6.3 Future work

Building upon the findings and recommendations presented in this study, several avenues for future research can contribute to a more comprehensive understanding of collaborative governance of excessive medical care. Firstly, empirical testing of the proposed evolutionary game model with real-world data from different regions and healthcare systems will validate and refine the regulatory strategies. Secondly, investigating the impact of technological advancements and healthcare policy changes on collaborative governance dynamics will provide insights for adaptive strategies. Additionally, exploring the role of public awareness and education in empowering patients to participate in governance actively can contribute to practical measures. Lastly, comparative studies across countries will identify global best practices for addressing excessive medical care. We can develop evidence-based strategies to promote reasonable medical treatment while mitigating excessive care risks by addressing these aspects.

## 7 Conclusion

The study explored the collaborative governance of excessive medical care using a three-way evolutionary game model involving the government regulatory department, medical institutions, and patients. The simulation analysis yielded valuable insights into the stakeholders' strategic interactions, highlighting the significance of patients' recognition of treatment outcomes and medical institutions' responsiveness to economic incentives and penalties. This study's application of evolutionary game theory to the challenge of overtreatment in healthcare marks a significant contribution to the field, offering a novel framework for understanding the complex dynamics between government, healthcare institutions, and patients, and providing practical, actionable insights for policymakers. Moreover, the innovative application of simulation analysis in this study not only enriches the academic discourse but also offers pragmatic insights for policy formulation aimed at curtailing excessive medical care, highlighting a novel pathway for bridging theoretical research with practical healthcare improvements.

Our research introduces a model that brings together government, healthcare providers, and patients to address healthcare governance. Moving forward, future studies could extend this model by incorporating additional stakeholders such as insurers and pharmaceutical firms, or by integrating real-world behavioral data to improve predictive accuracy. Policy-wise, our findings underscore the importance of dynamic regulatory frameworks that can adapt to evolving healthcare delivery environments, suggesting a need for continuous evaluation mechanisms and cross-sectoral coordination platforms to optimize collaborative governance in practice. By identifying strategies to reduce overtreatment and advocating for policies that encourage economic incentives and patient involvement, we offer insights into a more integrated approach to healthcare. This contribution modestly aims to provide a foundation for further studies and the development of policies aimed at improving the quality and efficiency of healthcare services.

However, it is essential to acknowledge the limitations of this study, as it relied heavily on theoretical foundations and simulated data. While the evolutionary game model provides a robust framework for understanding strategic interactions, it inevitably simplifies real-world dynamics. For example, the assumption of bounded rationality and homogeneous stakeholder behavior may overlook heterogeneity in institutional practices or patient decision-making processes. Furthermore, parameter values were derived from proportional estimations based on national-level statistics, which may not capture regional variations or institutional idiosyncrasies. These simplifications could introduce structural biases into the simulation outcomes and limit the generalizability of findings to other healthcare systems, particularly those outside the Chinese public hospital context. Future work should incorporate empirical calibration using granular, region-specific data and consider stochastic extensions of the model to better account for uncertainty and variability in stakeholder behavior. To enhance the credibility and applicability of the model, future research should prioritize empirical testing using real-world healthcare data. Researchers can validate and refine the proposed theoretical framework by incorporating actual data, enabling evidence-based policymaking and more effective healthcare regulatory measures. Although this study relies primarily on simulation-based analysis, all key parameters are transparently sourced and thoroughly tested for robustness. Future work will focus on empirical validation and refinement of the model using real-world data as they become available.

## Data Availability

The original contributions presented in the study are included in the article/supplementary material, further inquiries can be directed to the corresponding author.
